# The effectiveness of CBT-based daily supportive text messages in improving female mental health during COVID-19 pandemic: results from the Text4Hope program

**DOI:** 10.3389/fgwh.2023.1182267

**Published:** 2023-11-10

**Authors:** Raquel da Luz Dias, Reham Shalaby, Belinda Agyapong, Wesley Vuong, April Gusnowski, Shireen Surood, Andrew James Greenshaw, Vincent I. O. Agyapong

**Affiliations:** ^1^ Department of Psychiatry, Faculty of Medicine, Dalhousie University, Halifax, NS, Canada; ^2^ Department of Psychiatry, Nova Scotia Health Authority, Halifax, NS, Canada; ^3^ Department of Psychiatry, Faculty of Medicine and Dentistry, University of Alberta, Edmonton, AB, Canada; ^4^ Addiction and Mental Health, Alberta Health Services, Edmonton, AB, Canada

**Keywords:** mental health, female, COVID-19, text messages, Text4Hope

## Abstract

**Introduction:**

The COVID-19 pandemic has significantly exacerbated gender disparities in mental health, particularly impacting women. To address this, Alberta, Canada, launched Text4Hope, a Cognitive Behaviour Therapy-based text messaging intervention, to provide support and resources for psychological challenges during the pandemic. This study aimed to assess the effectiveness of Text4Hope in reducing stress, anxiety, depression, sleeping disturbances, and suicidal ideation among female subscribers during the COVID-19 pandemic.

**Methods:**

The study employed both an uncontrolled longitudinal design and a controlled cohort design. The uncontrolled longitudinal study analyzed a one-year dataset (*n* = 9,545) of clinical outcomes, comparing mean differences in mental health symptoms from baseline to 6 weeks after subscription. The controlled cohort design compared two groups, with (*n* = 1,763) and without (*n* = 567) intervention exposure during the same period. Data were collected through self-administered online surveys completed at baseline and six weeks after subscription. Sociodemographic information and validated scales (e.g., 10-item Perceived Stress Scale (PSS-10), Generalized Anxiety Disorder (GAD-7), and Patient Health Questionnaire (PHQ-9)) were used to assess mental health outcomes.

**Results:**

The results from the longitudinal study indicated a significant reduction in anxiety prevalence and anxiety symptoms, with a 19.63% decrease in GAD-7 mean score and a 32.02% decrease in likely anxiety from baseline to six weeks. Depressive symptoms and perceived stress also showed a significant reduction (*p* < 0.001), albeit to a lesser extent. In the controlled cohort study, the intervention group had significantly (*p* < 0.001) lower PHQ-9 [19.5 (SD 7.05)], GAD-7 [7.5 (SD 5.27)], and CMH [35.53 (SD 18.45)] scores. Additionally, the study found substantial differences (*p* < 0.001) in suicidal ideation (26.1 vs. 15.7) between groups but no significant differences in sleep disruption.

**Discussion:**

These findings suggest that Text4Hope could be an effective intervention for reducing stress, depression, suicidal ideation, and particularly anxiety symptoms among women during public emergencies. The study provides valuable insights into the potential benefits of text messaging interventions in supporting mental health during crisis situations.

## 1. Introduction

The COVID-19 pandemic has profoundly impacted women's mental health since it has brought to light pre-existing inequities and exacerbated disparities surrounding the psychological burden experienced by the female population (1). In pre-pandemic times, women were more likely to experience mental health problems. The prevalence of anxiety disorders in Canadian women is 6.3%, while in men, these figures reach 3.7% (2). Depression, which is a major risk factor for suicide, is two times more present in females than males (3), and although the prevalence of deaths caused by suicide is three times higher in males than in females (4), suicide attempts are 1.5 to 2 times more frequent in women and girls than men and boys (5). Women also report higher levels of perceived stress than men, which is generally attributable to their multiple roles and responsibilities in their careers and family (6). This is supported by data from the Canadian Community Health Survey, which has demonstrated a rising trend of high stress in the female population since 2003, with a rate of 23.7% for women in 2014 compared to 22.2% for males (7). High levels of stress can cause sleep problems, which have been found to be more common in women, particularly those with lower education and income, with a 10% higher prevalence than the male population (8).

Some of the factors that contribute to women's higher risk of mental health symptoms include biological and hormonal changes throughout life, particularly during pregnancy, the post-partum period, and menopause, which are considered critical periods (9, 10). Social and cultural pressures imposed on women, multiple roles and responsibilities within work and family, and a higher likelihood of experiencing poverty, traumatic events, discrimination, harassment, and gender-based violence can all play a role in the increased psychological burden on the female population (11). The gender disparities also appear to be influenced by women's greater natural tendency to report emotional symptoms and seek help than men, making them more likely to be diagnosed with mental health problems (12).

The uncertainty of the pandemic, public health measures (i.e., quarantine and lockdowns), and social disruptions of this unprecedented time have burdened everyone's mental health (13). Females were again at a higher risk of developing psychiatric symptoms during the COVID-19 outbreak (14). Amidst this public health emergency context, being a woman at a younger age, having student status and past psychiatric history, experiencing quarantine and being a COVID-19-positive patient (15) were all identified as risk factors for stress, anxiety, and depression (15, 16). Job status was also related to increased mental health symptoms, particularly for unemployed and female healthcare workers (17, 18, 19). Environmental aspects such as frequent social media use, pandemic news exposure, and an unhealthy diet were positively associated with greater odds of anxiety during the pandemic (20, 21). Furthermore, as observed in previous crises, the pandemic led to a surge in intimate partner violence and mothers witnessing violence against their children, which increased gender-based psychological issues (22–24).

To ease the strain on women's mental health, text messaging interventions are being proposed as a cost-effective program to provide support and resources to help them cope with psychological challenges, particularly those who may have limited access to traditional mental health services (25, 26). Text message interventions, also known as SMS interventions, are a type of digital mental health support that can be used for a variety of purposes, including supportive text messages, social support engagement, care team contact capabilities, data feedback, psychoeducation, adherence-based psychotherapy, remote care delivery, secure medication storage, and contingency planning (27, 28). Cognitive Behavioural Therapy (CBT), a psychotherapy approach that has been found effective for a wide range of mental health conditions, can be delivered in a variety of modalities (29). High-intensity CBT is generally provided by trained health professionals and commonly delivered in a face-to-face setting, individually or in a group format. Conversely, low-intensity CBT primarily focuses on self-help approaches and can be delivered through various platforms, including the Internet, telephone, and text messaging (30, 31). CBT modalities appear to be equally effective, and although the evidence on low-intense CBT interventions is not as extensive as for traditional CBT, the literature suggests that they can be just as effective as traditional CBT, particularly for individuals who may have limited access to traditional mental health services (30). Text message interventions have been found to be effective in reducing symptoms of anxiety, depression and perceived stress in women (32). Although highly beneficial, text message interventions cannot replace professional help but serve as a complementary tool to support mental health (33).

Text4Hope is a CBT-based text message intervention targeting individuals experiencing stress, anxiety and depression as a result of the COVID-19 pandemic (34). The service was launched in March 2020 in Alberta, Canada, and as of July 2021, over 54,000 Canadians had signed up for the Text4Hope program (35). Previous studies (36–38) have shown that the Text4Hope intervention can reduce symptoms of anxiety, depression, and perceived stress in the general population, with consistent results across different evaluation methodologies. A recent study using a two-pronged design to evaluate the Text4Hope database focused on youth mental health and confirmed the positive impact of the intervention by comparing outcomes over time and between groups (39). However, the specific impact of the program on female subscribers is not well understood, despite the majority of Text4Hope subscribers being women and the higher likelihood of women experiencing mental health issues. Therefore, this study aims to analyze a subset of the Text4Hope database to evaluate the intervention's efficacy in reducing symptoms of stress, anxiety, depression, sleep disturbances, and suicidal thoughts among female subscribers. By utilizing a longitudinal and a controlled cohort evaluation approach, this research provides insight into the potential benefits of text messaging interventions targeting female populations and their impact on mental wellbeing (33).

## 2. Methodology

### 2.1. Study design

**Figure 1:**
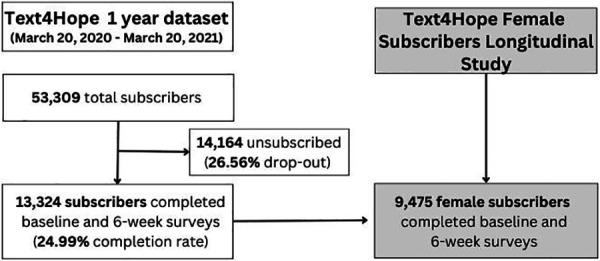
Longitudinal study flowchart and context of the female population dataset within the Text4Hope initiative.

This study utilized a specific dataset of Text4Hope female subscribers to evaluate the impact of the intervention on mental health outcomes of this particular population. It employed an uncontrolled within-subject comparison (longitudinal study) and a controlled between-subject comparison (cohort study) to analyze the available data. The longitudinal study examined clinical outcomes from a one-year dataset of female subscribers who completed both the baseline and the follow-up survey at 6 weeks after subscription. This group of Text4Hope subscribers corresponds to 24.99% (*n* = 9,475) of the 53,309 female individuals who subscribed to the program between March 2020 and March 2021. (Figure 1).

**Figure 2:**
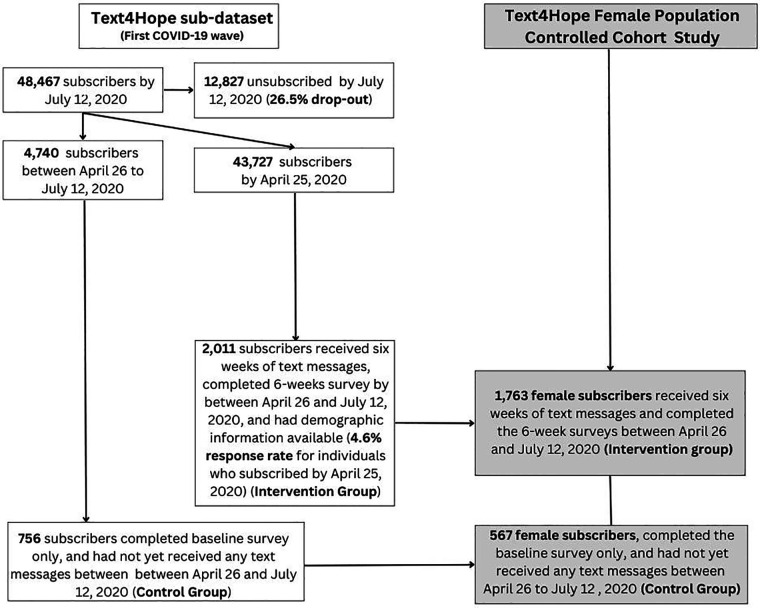
Controlled cohort study flowchart context of the female population dataset within the Text4Hope initiative.

The cohort study compared two distinct study populations of Text4Hope female subscribers (Figure 2). The first group, called the intervention group (IG, *n* = 1,723), comprised female subscribers who received the intervention for six weeks and completed the follow-up assessment survey six weeks after enrolment, between April 26 and July 12, 2020. The second group, referred to as the control group (CG, *n* = 567), consisted of female subscribers who joined the program in the same period, completed the baseline evaluation only, and had not yet received any text messages, as they were at the beginning of the program. Data from these subscribers (CG) were used solely to establish a comparison group of individuals who had not received the intervention at that point in time. By comparing participants with different levels of exposure to the intervention (IG with intervention exposure and CG without intervention exposure) during the same time period, we were able to evaluate the additional impact of the intervention on symptom levels beyond what would be expected from the natural course of the pandemic. This study design allowed us to examine the natural evolution of the outcomes of interest by observing individuals and comparing outcomes between groups. Both IG and CG were also part of the longitudinal study since a one-year dataset was included in the longitudinal analysis. However, the two studies are separate and distinct, each serving different purposes in evaluating the intervention's impact on mental health outcomes. The research protocol (32) was approved by the University of Alberta Research and Ethics Board (Pro00086163).

It is important to note that the primary goal of the Text4Hope program was to provide mental health support to individuals during the challenging times of the COVID-19 pandemic. As a result, the flowchart presented in this study reflects the natural progression of participants who voluntarily engaged with the program and completed the assessment surveys.

### 2.2. Study intervention

The intervention tested in this study was Text4Hope, a CBT-based text messaging program implemented in Alberta, Canada, during the COVID-19 pandemic. During this challenging time, the Text4Hope program emerged as a unique initiative, potentially contributing to reducing the gap to access mental health support since it was launched in March 2020. To the best of our knowledge, no other program of its kind was implemented in such a timely manner worldwide at that time.

The text messages provided by this program are based on CBT principles and were created by psychiatrists, psychologists, and mental health therapists to provide subscribers with bite-sized pieces of information about the cognitive triangle (thoughts, feelings, and actions) to achieve therapeutic benefit in the long run. The content of the text messages was designed to promote self-care, social support, hope, and affirmation, as well as to assist subscribers in managing symptoms of stress, anxiety and depression. Examples of text messages sent were:

• Self-care is always important. During times of stress, it becomes even more important as stress can take a toll on our mental and physical well-being. Hydrate, make a plan, prioritize sleep, meditate, unload and say no, connect with friends and family on phone even as you social distance.
• Notice when you're feeling sad, angry, lost or overwhelmed about life changes. Don't push the feelings away -acknowledge these feelings and let yourself grieve.
• When your emotions are high, calm yourself down by taking slow, deep breaths in through your nose and out through your mouth.

Individuals from the general population could self-subscribe to receive SMS text messages from the program for three months by texting the word “COVID19HOPE” to a short code number. The one-way (no-reply) daily supportive text messages were set to be automatically sent at 9 a.m. (Mountain Time) daily for the length of the program (3 months). After subscribing to the program, users were greeted with a welcome message and directed to a link where they completed the baseline assessment survey. Six weeks after enrollment, the link to the follow-up survey was also sent via SMS. Subscribers received the daily text messages regardless of whether or not they answered the surveys since participation was entirely voluntary.

### 2.3. Data collection

Study data were collected from self-completed online surveys powered by REDCap, including demographic information and mental health symptoms, by Text4Hope's subscribers. Demographic information, including age, ethnicity, educational level, employment, relationship and housing status, were collected at baseline. Validated scales that reflect the mental health outcomes of interest in the general population were used to assess subscribers' mental health symptoms at baseline and six weeks after enrollment. Originally, Text4Hope follow-up assessments were conducted six weeks and three months after the baseline assessment, covering the entire program length. However, in this paper, we decided to focus on the six-week follow-up only, aiming to capture initial outcomes into the early impact of the Text4Hope program on the mental health outcomes of female subscribers.

Data for the observational controlled study were collected from April 26 to July 12, 2020. This period was chosen because the majority of subscribers joined Text4Hope in the first three months of the program. Having a large number of individuals who subscribed to the program in the same time frame allowed investigators to ensure that both the IG and CG were exposed to similar external factors and contextual influences. In addition, this duration represents the timeframe of the follow-up period for the first study cohort, who were recruited in the initial phase (from March 23 and April 25, 2020), and it is worth noting that the only difference between the IG and CG was that the CG had subscribed to the Text4Hope program and completed the baseline assessment, without receiving yet any intervention. This period also coincided with the first wave of the pandemic, and with the first lockdown in Alberta that began on March 17th, 2020. In contrast, the longitudinal study dataset was collected over a year, starting from March 20, 2020, and ending on March 20, 2021, covering the entire first year of the pandemic. During this period, the lockdown restrictions in Alberta were gradually lifted between May and July 2020, although some limitations remained in place until July 12, 2020. The province experienced a second wave in the fall of 2020, which prompted a new round of restrictions and closures (40).

### 2.4. Outcome measures

Study outcomes were measured through quantitative data from self-completed online surveys. Perceived stress, symptoms of anxiety and depression were assessed by using the 10-item Perceived Stress Scale (PSS-10) (41), the Generalized Anxiety Disorder (GAD-7) scale (42), and the Patient Health Questionnaire (PHQ-9) (43), respectively. The primary outcome of the study was determined by analyzing the total scores of each scale. This involved assessing the mean changes between baseline and 6 weeks in the longitudinal study, and calculating the mean difference (95% confidence interval) between the IG and CG groups in the controlled study. Secondary outcomes were differences between baseline and 6 weeks and between groups IG and CG in self-reported prevalence rates of moderate/high stress, likely generalized anxiety disorder GAD, and likely major depressive disorder (MDD). The criteria for moderate/high stress was established as having a PSS-10 score of 14 or higher (44). The presence of a GAD-7 score of 10 or higher was indicative of GAD (42), while a PHQ-9 score of 10 or higher was suggestive of MDD (43). The Composite Mental Health (CMH) score, which is the sum of all scores from the previously mentioned scales, was also assessed. The third and ninth questions of PHQ-9 were used to evaluate sleep disturbances and suicidal ideation, respectively.

### 2.5. Hypothesis

The hypothesis for this study is that after six weeks of receiving the daily supportive text messages (IG), female participants would have at least 25% lower scores on the CMH score, PSS-10, PHQ-9, and GAD-7 scales, and the prevalence for each of moderate/high stress, likely GAD, likely MDD, disturbed sleep and suicidal ideation and thoughts of self-harm would also be lower for IG participants than for CG participants. This conservative forecast is based on previous randomized controlled trials (44, 45) utilizing supportive text messages for patients with major depressive disorder and alcohol use disorder. These studies found a 25% and a 50%, respectively, greater reduction in their depressive symptom scores compared to patients who did not receive the daily supportive messages.

### 2.6. Statistical analysis

The longitudinal study compared sociodemographic and clinical factors between participants who completed both baseline and sixth-week surveys and those who completed only the baseline survey using the Chi-square. Descriptive statistics were used to analyze the distribution of demographic characteristics and isolation conditions of the studied population. We employed a paired sample t-test and the McNemar test to compare mean PSS-10, GAD-7, PHQ-9, and CMH scores and the prevalence of moderate to high stress, likely GAD, and likely MDD from baseline to six weeks for individuals who completed questionnaires at both periods. The controlled study compared IG with CG demographics. A Chi-Square test was used to compare the prevalence of moderate to high stress, likely depression, likely anxiety, having suicidal ideation and sleep disturbance symptoms between the IG at 6 weeks and CG at baseline. In addition, mean scores were compared using independent t-tests, and Welch t-test was used when equality of variance assumption was violated as indicated by Leven's test. To address the issue of multiple testing and potential inflation of type I error, the Bonferroni correction was applied. Finally, multivariate logistic regression models were performed to assess the impact of the intervention on the clinical variables while controlling for demographic variables. There were no imputations for missing values and no matching techniques for the demographic characteristics between the two study groups was performed. Participants were included in the analysis even with only one outcome variable. In other words, individuals who had missing data for one outcome measure but had data available for other outcome measures were still included in the analysis for the respective measures they had completed. This accounts for the differences in the sample size (*n*) for each variable, as it may vary from the initial sample size included in the sociodemographic analysis. However, we controlled for these characteristics in a logistic regression model, as previously stated. Data analysis was performed using SPSS for Windows version 25 (IBM Corporation) (46).

## 3. Results

### 3.1. Longitudinal study results

The longitudinal study illustrates results from participants who completed the baseline and 6-week surveys. Sociodemographic characteristics of the longitudinal study participants are shown in Table 1. A total of 9,475 female participants subscribed to Text4Hope during a one-year timeframe of the study. The majority of the studied population was between 26 and 60 years old (7,499, 79.1%), white (7,825, 83.1%), had postsecondary education (7,752, 86.7%), was employed (6,021, 67.3%), was married, cohabiting or partnered (6,337, 70.9%), and owned a house (5,894, 66.8%). Among females aged 25 years or less, we found a higher frequency of students (349, 36.2%), singles (440, 45.8%) and individuals living with family (553, 58.3%).

**Table 1:** Distribution of demographic characteristics of female subscribers who completed baseline and 6-week surveys, based on the age category of the participants.

Variables	≤25 y	26–40 y	41–60 y	>60 y	Total
	*n* (%)	*n* (%)	*n* (%)	*n* (%)	*n* (%)
	1,019 (10.8)	3,355 (35.4)	4,144 (43.7)	957 (10.1)	9,475 (100)
Ethnicity					
White	731 (72.2)	2,710 (81.1)	3,535 (85.7)	849 (90.1)	7,825 (83.1)
Indigenous	49 (4.8)	142 (4.2)	137 (3.3)	21 (2.2)	349 (3.7)
Asian	95 (9.4)	212 (6.3)	154 (3.7)	11 (1.2)	472 (5.0)
Other^a^	138 (13.6)	278 (8.3)	298 (7.2)	61 (6.5)	775 (8.2)
Education level					
Less than high school diploma	198 (20.6)	62 (1.9)	46 (1.2)	13 (1.4)	319 (3.6)
High school diploma	171 (17.8)	244 (7.6)	284 (7.3)	98 (10.9)	797 (8.9)
Postsecondary education	585 (60.8)	2,859 (89.5)	3,527 (90.8)	781 (86.9)	7,752 (86.7)
Other education^a^	8 (0.8)	31 (1.0)	28 (0.7)	7 (0.8)	74 (0.8)
Employment status					
Employed	374 (38.8)	2,315 (72.5)	3,015 (77.6)	317 (35.1)	6,021 (67.3)
Homemaker	10 (1.0)	287 (9.0)	180 (4.6)	18 (2.0)	495 (5.5)
Unemployed	190 (19.7)	379 (11.9)	434 (11.2)	54 (6.0)	1,057 (11.8)
Retired	0 (0.0)	0 (0.0)	147 (3.8)	490 (54.3)	637 (7.1)
Student	349 (36.2)	119 (3.7)	29 (0.7)	2 (0.2)	499 (5.6)
Other^a^	40 (4.2)	95 (3.0)	81 (2.1)	22 (2.4)	238 (2.7)
Relationship status					
Married/cohabiting/partnered	497 (51.8)	2,447 (76.5)	2,839 (73.1)	554 (61.4)	6,337 (70.9)
Separated or divorced	8 (0.8)	124 (3.9)	456 (11.7)	148 (16.4)	736 (8.2)
Widowed	0 (0.0)	7 (0.2)	53 (1.4)	91 (10.1)	151 (1.7)
Single	440 (45.8)	597 (18.7)	514 (13.2)	101 (11.2)	1,652 (18.5)
Other^a^	15 (1.6)	24 (0.8)	21 (0.5)	8 (0.9)	68 (0.8)
Housing status					
Own a home	84 (8.9)	1,890 (59.9)	3,158 (82.3)	762 (86.3)	5,894 (66.8)
Living with family	553 (58.3)	222 (7.0)	78 (2.0)	14 (1.6)	867 (9.8)
Renting	302 (31.8)	1,011 (32.0)	555 (14.5)	96 (10.9)	1,964 (22.3)
Other^a^	10 (1.1)	34 (1.1)	45 (1.2)	11 (1.2)	100 (1.1)

^a^ "Other” is meant to cover categories not included in the survey options.

Table 2 demonstrates the changes in baseline mean scores for the primary outcome variables after the introduction of Text4Hope. The table results indicate that mean scores for PSS-10, PHQ-9, and GAD-7 were lower after six weeks of receiving text messages. Specifically, there was a reduction of 6.24%, 8.80%, and 19.63% in mean scores, respectively (relative measure of change). This difference was significant for all three variables (*p* < 0.001), with a stronger effect size for anxiety.

**Table 2:** Changes in baseline mean scores of PSS-10, PHQ-9, and GAD-7 after the introduction of Text4Hope.

Measure	Scores			Change from baseline %	Mean difference (95% CI)	*P*-value	*t*-value	Effect size^a^
	*n*	Baseline mean (SD)	6-week mean (SD)					
PSS-10 total score	1,043	20.81 (6.86)	19.51 (7.13)	6.24	1.30 (0.96–1.63)	<.001	7.58	0.19
PHQ-9 total score	963	9.31 (6.03)	8.49 (5.70)	8.80	0.81 (0.52–1.10)	<.001	5.46	0.14
GAD-7 total score	931	9.42 (5.62)	7.57 (5.24)	19.63	1.85 (1.56–2.13)	<.001	12.57	0.34

^a^ Cohen's d.

Table 3 shows the percentage of female subscribers who reported moderate-to-high stress, likely depression, and likely anxiety at baseline and 6 weeks. A significant reduction (*p* < 0.001) in the prevalence of moderate-to-high stress (86.5 vs. 79.5), likely depression (42.5 vs. 36.4), and likely anxiety (45.6 vs. 31.0) were found when comparing data from both time points.

**Table 3:** Prevalence of moderate-to-high stress, likely GAD, and likely MDD at baseline and 6-weeks (McNemar test).

Clinical Condition	Prevalence *n/N* (%)		Change from baseline %	df	*p* value
	Baseline	Six-weeks			
Moderate-to-High Stress^a^	902/1,043 (86.5)	829/1,043 (79.5)	8.09	1	<.001
Likely MDD^b^	409/963 (42.5)	351/963 (36.4)	14.35	1	<.001
Likely GAD^c^	425/931 (45.6)	289/931 (31.0)	32.02	1	<.001

^a^ Moderate or High Stress defined as PSS-10 ≥ 14.

^b^ Likely GAD defined as GAD-7 ≥ 10.

^c^ Likely MDD defined as PHQ-9 ≥ 10.

### 3.2. Controlled cohort study results

The results of the controlled trial exhibit data from two distinct groups of Text4Hope female subscribers (IG with intervention exposure and CG without intervention exposure). Table 4 presents the demographic characteristics of IG (*n* = 1,763) and CG (*n* = 567) at the time of their enrollment in the program. Similar majorities (41–60 years old, white, post-secondary education, employed, married, and homeowners) were found in both groups. However, the IG and CG had significant variations (*p* < 0.001) in age, educational level, employment status, and housing status.

**Table 4:** Sociodemographic distribution characteristics of female subscribers of the IG and CG at baseline.

Variable	CG *n* (%)	IG *n* (%)	Total *n* (%)	*χ* ^2^	*P* value
Age					
≤ 25 y	78 (13.8)	151 (8.6)	229 (9.8)	15.27	0.002
26–40 y	166 (29.3)	489 (27.7)	655 (28.1)		
41–60 y	257 (45.3)	890 (50.5)	1,147 (49.2)		
> 60 y	66 (11.6)	233 (13.2)	299 (12.8)		
Ethnicity					
White	461 (80.3)	1,475 (83.9)	1,936 (83.1)	6.65	0.084
Indigenous	28 (4.9)	53 (3.0)	81 (3.5)		
Asian	29 (5.1)	89 (5.1)	118 (5.1)		
Other^a^	56 (9.8)	140 (8.0)	196 (8.4)		
Education level					
Less than a high school diploma	35 (6.1)	35 (2.4)	70 (3.4)	29.45	<.001
High school diploma	56 (9.7)	102 (6.9)	158 (7.7)		
Post-secondary	477 (82.5)	1,337 (90.2)	1,814 (88)		
Other^a^	10 (1.7)	9 (0.6)	19 (0.9)		
Employment status					
Employed	400 (69.3)	1,053 (71.6)	1,453 (71)	157.66	<.001
Unemployed	42 (7.3)	174 (11.8)	216 (10.6)		
Retired	10 (1.7)	149 (10.1)	159 (7.8)		
Student	118 (20.5)	70 (4.8)	188 (9.2)		
Other^a^	7 (1.2)	24 (1.6)	31 (1.5)		
Relationship status					
Married/cohabiting/partnered	400 (69.3)	979 (66)	1,379 (66.9)	6.25	0.181
Separated or divorced	42 (7.3)	154 (10.4)	196 (9.5)		
Widowed	10 (1.7)	37 (2.5)	47 (2.3)		
Single	118 (20.5)	299 (20.2)	417 (20.2)		
Other^a^	7 (1.2)	14 (0.9)	21 (1)		
Housing status					
Own a home	351 (62)	1,027 (70)	1,378 (67.7)	17.78	<.001
Live with family/friend	73 (12.9)	132 (9)	205 (10.1)		
Renting	131 (23.1)	299 (20.4)	430 (21.1)		
Other^a^	11 (1.9)	10 (0.7)	21 (1)		

^a^ "Other” is meant to cover categories not included in the survey options.

Table 5 summarizes the differences between the intervention and control groups in the three primary outcome variables and the Composite Mental Health (CMH) score. With the exception of PSS-10, the intervention group had significantly (p < 0.001) lower PHQ-9 [19.5 (SD 7.05)], GAD-7 [7.5 (SD 5.27)], and CMH [35.53 (SD 18.45)] scores. Bonferroni correction was applied for multiple testing, demonstrating a significance level of *p* 0.012, which maintains the significance level for the CMH score.

**Table 5:** Independent sample t-test comparing the mean scores for IG and CG on PSS-10, the GAD-7, and PHQ-9 scales and the Composite Mental Health (CMH) score.

Measure	Scores				Mean difference (95% CI)	*P* value*	*t*-value (df)	Effect size
	*n*	IG mean (SD)	*n*	CG, mean (SD)				
PSS-10 total score	1,636	19.50 (7.05)	535	22.50 (7.30)	3.00 (2.30–3.70)	<.001	8.46 (2,169)	0.42
PHQ-9 total score	1,525	8.52 (5.84)	502	11.08 (6.70)	2.56 (1.95–3.17)	<.001	8.20 (2,025)	0.42
GAD-7 total score	1,493	7.50 (5.27)	489	9.64 (6.08)	2.14 (1.58–2.70)	<.001	7.50 (1,980)	0.39
CMH Score	1,489	35.52 (16.70)	487	43.30 (18.45)	7.78 (6.02–9.54)	<.001	8.70 (1,974)	0.45

CI, confidence interval; CMH, Composite Mental Health Score; Effect Size: Hedge's g.

* Welch *t*-test was used.

Table 6 demonstrates the difference in the prevalence of moderate to high stress, likely depression, likely anxiety,sleep disturbance, and suicidal ideation/thoughts of self-hams between the IG and CG. The intervention group had significantly (*p* < 0.001) lower moderate to high stress (88.6 vs. 79.0), likely depression (51.4 vs. 36.2), likely anxiety (47.0 vs. 31.0), and suicidal thoughts (26.1 vs. 15.7). No differences were detected in the prevalence of sleep disturbance (*p* > 0.005) between the two groups.

**Table 6:** Chi-square test of association between prevalence of clinical parameters and study arm.

Clinical Condition	Prevalence *n/N* (%)		*χ*2 (df)	*p* value	Effect size (Cramer's V.)
	IG	CG			
Moderate-to-High Stress^a^	1,293/1,636 (79.0)	474/535 (88.4)	24.35 (1)	<.001	0.106
Likely MDD^b^	552/1,525 (36.2)	258/502 (51.4)	36.36 (1)	<.001	0.134
Likely GAD^c^	463/1,493 (31.0)	230/489 (47.0)	41.60 (1)	<.001	0.145
Sleep disturbance^d^	1,177/1,525 (77.2)	408/502 (81.3)	3.71 (1)	0.054	0.043
Suicidal ideation/thoughts of self-harm^e^	240/1,525 (15.7)	131/502 (26.1)	27.10 (1)	<.001	0.116

^a^ Moderate or High Stress defined as PSS-10 ≥ 14.

^b^ Likely GAD defined as GAD-7 ≥ 10.

^c^ Likely MDD defined as PHQ-9 ≥ 10.

^d^ Evaluated through the third question of PHQ-9.

^e^ Evaluated through the ninth question of PHQ-9.

Table 7 explores the predictive value of each group (with or without intervention) on the likelihood of the psychological conditions of interest when controlling for demographic (age, ethnicity, education level, relationship, employment and housing status). Statistical significance was observed in the models predicting the moderate/high stress (*Χ*^2^ (df = 21; *n* = 1,896) = 149.36, *p* < .001, accounting for 7.6% (Cox and Snell R^2^) to 12.3% (Nagelkerke R^2^) of the variance; and correctly classified 81.5% of the cases), likelihood of MDD (*Χ*^2^ (df = 21; *n* = 1,813) = 165.11, *p* < .001, accounting for 8.7% (Cox and Snell R^2^) to 11.7% (Nagelkerke R^2^) of the variance; and correctly classified 65.3% of the cases), likelihood of GAD (*Χ*^2^ (df = 21; *n* = 1,774) = 190.78, *p* < .001, accounting for 10.2% (Cox and Snell R2) to 14% (Nagelkerke R^2^) of the variance; and correctly classified 69.1% of the cases), and suicidal ideation/self-harm thoughts (*Χ*^2^ (df = 21; *n* = 1,813) = 190.80, *p* < .001, accounting for 10% (Cox and Snell R^2^) to 16.2% (Nagelkerke R^2^) of the variance; and correctly classified 83.1% of the cases). The model predicting sleep disruption (*Χ*^2^ (df = 21; *n* = 1,813) = 30.30, p.086, accounting for 1.7% (Cox and Snell R^2^) to 2.6% (Nagelkerke R^2^) of the variance; and correctly classified 78.6% of the cases) was not statistically significant. Results from the table show that the intervention group had lower odds of having the psychological symptoms (*p* < 0.001 and OR < 1), meaning at the Text4Hope intervention was a significant predictor for lower odds of moderate to high stress (OR 0.54), likely anxiety (OR 0.55), likely depression (OR 0.57), and suicidal ideation (OR 0.55). The text messaging intervention was not found to be a predictor for lower sleep disturbances (*p* > 0.05 and OR 0.80).

**Table 7:** Summary from five multivariate logistic regression models for respondents’ likelihood to present with moderate to high stress, likely anxiety, likely depression, suicidal ideation or self-harm thoughts, and sleep disturbance symptoms, with a focus on the type of subscribers (intervention or control), while controlling for other demographic variables.

Outcome measure	*p*-Value	Odds ratio	95% CI for OR	
			Lower	Upper
Moderate/High-Stress^a^	<0.001	0.54	0.39	0.75
GAD likely^b^	<0.001	0.55	0.43	0.70
MDD likely^c^	<0.001	0.57	0.45	0.72
Experienced Suicidal Ideation/Self Harm Thoughts^d^	<0.001	0.55	0.41	0.73
Experienced Sleep Disturbances^e^	0.121	0.80	0.61	1.06

^a^ Moderate or High Stress defined as PSS-10 ≥ 14.

^b^ Likely GAD defined as GAD-7 ≥ 10.

^c^ Likely MDD defined as PHQ-9 ≥ 10.

^d^ Evaluated through the third question of PHQ-9.

^e^ Evaluated through the ninth question of PHQ-9.

## 4. Discussion

This observational study examined the effectiveness of Text4Hope in improving mental health among female subscribers during the COVID-19 pandemic. The overall results of both longitudinal and controlled studies suggest that Text4Hope intervention may be effective in reducing symptoms of stress, depression, and anxiety among female subscribers who engaged in the program and completed the assessment surveys.

Females are more likely to engage in different e-health behaviours than males (47), and they usually present higher satisfaction levels as text messaging program users than their male counterparts (48, 49). This partially explains why most Text4Hope's subscribers are female, accounting for approximately 88% of the total subscribers according to data from previous studies (36–38). The longitudinal study revealed demographic characteristics of a privileged segment of the population: they are between the ages of 26 and 60, white, have completed some college-level education, are currently employed, are in a relationship, and own their own home. The low prevalence of marginalized women groups, such as women from ethnic or racial minorities in the study population, may be due to the trend of this group having less access to mobile technology than the general population, suggesting that access to the Text4Hope program may also be limited (50). Age, level of education, income, and geographic location are all factors that contribute to less mobile device accessibility. Nonetheless, these disadvantaged women groups may encounter additional barriers to mobile technology access, such as a lack of digital literacy, infrastructure, or discrimination (51, 52). Inequalities in the use of e-health services are likely to persist unless efforts are made to increase access to mobile phones, especially among the underserved female population, as part of implementing population-level digital health programs.

The sociodemographic characteristics of female subscribers were analyzed in a controlled trial, revealing significant differences in age, educational level, employment status, and housing status between the Intervention Group (IG) and Control Group (CG). These differences might be related to the time taken by each group to seek mental health support during the pandemic. Specifically, individuals in the IG (who had completed the 6-week survey during the data collection period) began seeking mental health support through Text4Hope just one week after the World Health Organization (WHO) officially announced the pandemic on March 11, 2020. In contrast, the CG (who had enrolled in the program during the data collection period) began seeking mental health support almost two months later. Our findings suggest that subscribers younger than 25 years old, had lower education levels, and were living with family tended to take longer to seek help. On the other hand, women between the ages of 41 and 60, with higher education levels, who were unemployed and owned a home tended to seek help faster during this public emergency. These findings highlight the importance of considering sociodemographic factors when designing interventions for mental health support during crises to ensure that vulnerable populations receive timely support.

Although the results of this study did not entirely corroborate the expected changes stated in the researchers' hypothesis, the intervention had a significant (*p* < 0.001) influence on the primary outcomes of the study, reducing perceived stress, anxiety, and depressive symptoms. Two randomized controlled trials using CBT-based text messaging programs as the intervention to be tested have yielded comparable results (44, 45). From baseline to six weeks, there was a reduction of −19.63% in the GAD-7 mean score, indicating a greater change in anxiety symptoms. Changes related to depressive symptoms and perceived stress were less expressive but still significant. The same trend was found in the prevalence of moderate-to-high stress, likely depression, and likely anxiety and in the mean scores of the three primary outcome variables at enrolment (BS) and six weeks (6 W) in the study. A significant (*p* < 0.001) decrease in the prevalence of these symptoms, especially in likely anxiety, and lower scores in the related assessment scales were found. It is worthy to note that the results were presented in both mean scores and prevalence rates of symptom categories to provide a comprehensive understanding of the impact of the intervention on mental health outcomes. By reporting mean scores, the magnitude of change in symptom severity can be assessed, while the prevalence rates enable the examination of the proportion of participants who experienced clinically significant symptom reduction. Together, these results do not provide additional evidence of the intervention's effectiveness but rather offer an alternative perspective on the outcomes. However, the differences encountered suggest that Text4Hope intervention successfully improved mental health outcomes among female subscribers in a short time frame of 6 weeks. Similar results were found in previous studies evaluating male population. We have previously evaluated the impact of the Text4Hope intervention in men and the results showed a positive impact of the intervention in this gender group.

CBT-based text messages from the Text4Hope program effectively deliver a low-intensity CBT intervention and promote mental well-being among a large population. The impact of these bite-sized CBT pieces may be related to the “just-in-time” nature of the intervention, delivering support at the moment and in the context the person needs (53). This is especially important during times of high vulnerability or periods of increased susceptibility to negative emotions and thoughts (54), such as the ones the population experienced during the pandemic's social isolation. The “time and place” component of daily supportive text messages appears to be critical in determining the beneficial support in this situation. Furthermore, the portable and ubiquitous nature of mobile phones allows individuals to continuously access mental health support, even in the face of numerous restrictions and other safety measures imposed on in-person services, which disrupted or halted critical mental health services in 93% of countries worldwide (55).

However, the unprecedented and uncertain nature of the pandemic context might have hindered the effectiveness of these CBT chunks in reducing perceived stress. Individuals' responses to stress and coping mechanisms influence the difficulty of addressing specific stressors during public health emergencies (56); therefore, a one-size-fits-all approach may not be effective for everyone. Tailoring text messages to the recipient's needs and preferences could increase their efficacy (57). Text4Support is an example of a CBT-based text messaging program tailored to address specific psychological problems through theme-specific content focused on the management of symptoms of specific mental health concerns (e.g., depression, anxiety, bipolar disorders) and messages of self-care, social support, hope, affirmation, and recovery. Moreover, it is important to consider that the engagement in e-Mental Health interventions (i.e., text messaging, mobile apps, etc) tends to decrease over time (58, 59). The high attrition rates and poor rates of sustained engagement might be seen as a limitation—and it is, considering long-term impacts or severe mental health issues (60)—but during emergencies like pandemics, such interventions can be promptly delivered, significantly reducing mental health symptoms for many individuals (37, 61). As such, they may serve as a valuable low-intensity intervention tool, acting as a first step in a stepped-care approach to service delivery (62), until other mental health interventions can be implemented to address the broader population's needs. Conversely, it is noteworthy to mention the findings from a study on Text4Hope subscribers' satisfaction (48). The study revealed that the majority of respondents (1,531/1,716, 89.2%) always read the text messages after six weeks of receiving them, with about 23.4% (401/1,716) of respondents indicating that they always or often returned to read the text messages.

It is essential to consider that the results associated with the Text4Hope intervention may not be exclusively attributed to the CBT-based text message intervention. The Hawthorne Effect, a psychological phenomenon where individuals alter their behavior or performance when aware of being observed (63), may also have played a role. However, researchers can employ strategies to assess whether the Hawthorne Effect influenced a study. In our study, we have incorporated specific features in our design that allow us to make informed inferences about the potential impact of the Hawthorne Effect on the findings. For example, in the controlled cohort study, we compared data from individuals who were at the baseline stage (not yet exposed to the intervention) with the changes observed in the intervention group. The presence of significant differences in outcomes indicates that the CBT-based text messages likely had a distinct impact on the subscribers. Furthermore, it is important to note that the Hawthorne Effect often diminishes over time as individuals become accustomed to being observed (64). In our longitudinal analysis, we did not observe this diminishing effect, as the results remained consistent after 6 weeks. By incorporating multiple study designs, our aim was to provide a comprehensive view of the intervention's effects, taking into account both immediate impacts and potential influences, such as the Hawthorne Effect, over an extended period. This approach enhances our understanding of the intervention's impact and its underlying mechanisms.

Finally, statistically significant differences were found in the prevalence of sleep disturbance between the IG and the CG, although CBT is a safe and highly effective treatment for sleep problems (65). Interestingly, the results showed significant differences between groups for suicidal ideation (*p* < 0.001), as well as a protective impact against the perceived stress, likely depression and anxiety, and suicidal thoughts, despite the sociodemographic characteristics of the subjects at baseline. Studies have shown that text-based interventions can be effective in reducing suicidal ideation and increasing positive thinking, particularly for the highly distressed, young female population, including typically underserved minorities and a substantial percentage of individuals who do not receive help elsewhere (66, 67). While text-based crisis hotlines can provide immediate support to individuals in crisis, Text4Hope can provide accessible and convenient ongoing support to individuals who are at risk for suicidal thoughts and can reduce the likelihood of certain psychological symptoms (68).

Several limitations must be acknowledged in this study. Self-selection and dropouts could bias the study results if they relate to the examined outcomes. Text4Hope subscribers may have been more motivated to improve their mental health and more likely to respond positively to the intervention, (i.e., reading the text messages and responding to the follow-up surveys) than those who did not enroll. This may have led to an overestimation of the effect of Text4Hope on mental health outcomes. On the other hand, severe mental health symptoms could be a reason for dropout, leading to an underestimation of Text4Hope's impact on mental health outcomes. However, the reasons for dropouts are unknown, and it is possible that those who did not complete the surveys may also have benefited from the intervention. The high rate of dropouts resulted in a reduced sample size, raising valid concerns about the potential impact on the study results. However, it is worth noting that we used all available data from those who voluntarily participated in the assessment surveys, which provides valuable insights into the effectiveness of the intervention among those who engaged with the program. It should be noted that the results may not be generalizable to the broader population who did not respond to the evaluation surveys. Seasonality might also be affected by the results of this study. The lifting of lockdown measures and the decrease in case numbers during the summer of 2020, followed by a decline in mental health symptoms during the same period, may have influenced the impact of the intervention.

The controlled cohort design also has some limitations due to the real-world nature of the Text4Hope program, which made it impractical to have a separate group of individuals without the intervention. Randomization and withholding the intervention were ethically inappropriate, as it could harm those in need of support during the pandemic. Despite potential bias, this design reflects the real-world setting and offers valuable insights into the intervention's effectiveness. The data collected during the same two-month period for both groups allow us to reasonably attribute any observed score differences to the intervention. Lastly, the corrections employed in the data analysis may not have adequately addressed violations of the normality assumption. However, this violation apparently has not impacted the produced results upon using parametric analysis (t-test). This is due to the large sample size included in this study which yields no problem when using this type of test. This implies that parametric procedures can be used even when the data are not normally distributed (69, 70). The major strength of this study was the robustness of its design, which included a controlled cohort that allowed the investigators to control for the impact of the natural course of the pandemic on mental health outcomes, and for self-selection bias, demonstrating that the intervention was effective regardless of these limitations.

In considering particularities of women's health, we acknowledge the potential impact of sensitive periods, such as pregnancy, puerperium, or menopause on mental health symptoms in the female population. Therefore, further research is needed to explore the interplay between women's health and mental health outcomes during sensitive periods.

In conclusion, the findings of this study demonstrate the effectiveness of Text4Hope, a CBT-based daily supportive text messaging program, in improving mental health outcomes among women during the COVID-19 pandemic. E-mental health interventions, including text messaging programs like Text4Hope, have the potential to address the unique challenges and disparities in women's mental health by providing accessible mental health support. However, it is important to consider the gender gaps in technology usage and women's specific usage patterns before implementing strategies to increase uptake and maximize the benefits of such interventions.

## Data Availability

The raw data supporting the conclusions of this article will be made available by the authors, without undue reservation.
